# Bilateral Arthroscopic Superior Capsular Reconstruction of the Shoulder for Irreparable Rotator Cuff Tears: A Case Report and Description of Surgical Technique

**DOI:** 10.7759/cureus.17525

**Published:** 2021-08-28

**Authors:** Waleed Albishi, Khalid Murrad, Amr Elmaraghy

**Affiliations:** 1 Orthopaedic Surgery, College of Medicine King Saud University, Riyadh, SAU; 2 Orthopaedic Surgery, King Saud University, Riyadh, SAU; 3 Orthopaedic Surgery, University of Toronto, Toronto, CAN

**Keywords:** arthroscopy, upper limb, bilateral, rotator cuff, capsule reconstruction

## Abstract

The treatment strategies for rotator cuff tears have grown more sophisticated in recent years. Over the past decade, arthroscopic superior capsular reconstruction (SCR) has become popular for treating irreparable rotator cuff tears. Despite the popularity, the literature on the clinical outcomes of SCR is limited. Several surgical procedures using variable graft materials and different techniques have been proposed promising early clinical results with improvements in shoulder pain, range of motion, and overall function. In this paper, we present a case of bilateral massive irreparable rotator cuff tears, with a full description of our surgical technique and the successful outcome of our management.

## Introduction

Management techniques and algorithms for rotator cuff tears have grown more sophisticated in recent years [1]. Although some effective and useful non-surgical options have been successful for specific conditions, they can often manage persistently symptomatic tears with surgical repair [2]. Some massive, chronic, and retracted tears cannot be repaired, especially with poor tendons and muscle quality. Over the past decade, arthroscopic superior capsular reconstruction (SCR) has become popular for treating irreparable rotator cuff tears. A study has shown promising early results with improvements in pain, range of motion, and function [3]. This paper presents a case that has not been previously reported describing irreparable rotator cuff tears in an otherwise healthy gentleman who was successfully treated with bilateral arthroscopic superior capsular reconstruction.

## Case presentation

**Figure 1:**
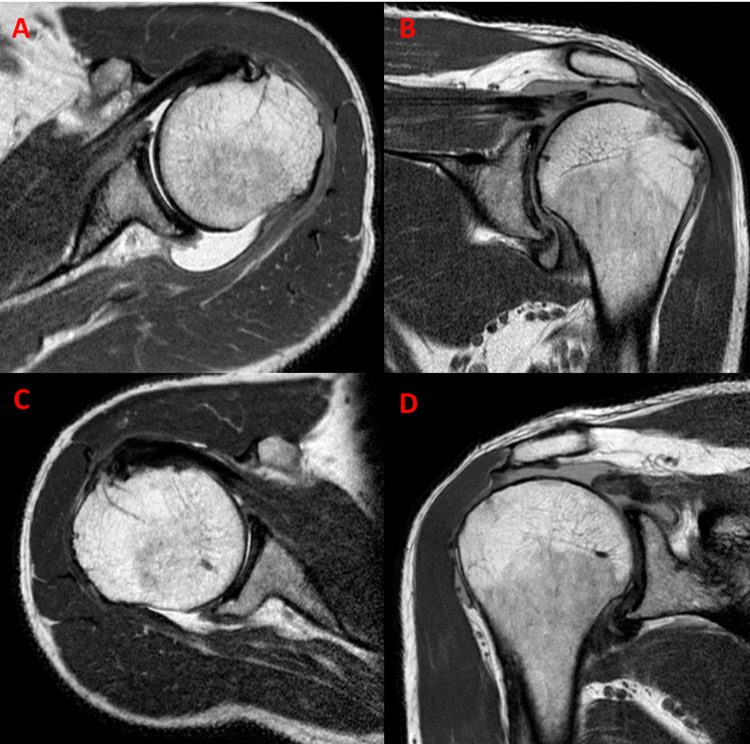
Bilateral shoulder MRI showing irreparable rotator cuff tear on axial and coronal images. Left shoulder (A, B), right shoulder (C, D).

This gentleman was a 50-year-old right-hand dominant self-employed driver who presented with a chief complaint of bilateral anterolateral shoulder pain which began subacutely and had persisted for over a year. It woke him up from sleep, and he had failed non-operative treatment, including physiotherapy. He was healthy otherwise and specifically was pre-diabetic (diet controlled) but a non-smoker. The clinical exam was notable for pseudoparalysis but good passive range of motion, albeit with subacromial crepitus and positive impingement signs. There was no tenderness of the AC joint but a positive Yergason’s sign for proximal LHB irritability. Significant weakness was noted when testing supraspinatus and infraspinatus. Subscapularis was tested as normal power. X-rays confirmed significant proximal migration of the humerus but no significant osteoarthritic changes. An MRI showed some long head of biceps (LHB) edema and massive supra and infraspinatus tear with significant atrophy of the involved muscles with moderate fatty degeneration (Figure 1). Given that his symptoms were intolerable, he was consented to arthroscopic surgical exploration and attempted repair with the possible need for dermal allograft superior capsular reconstruction.

Surgery was performed on the right side first, followed by the left side 10 months later. At the time of both procedures, found the articular surface to have only grade 2 changes, and the LHB was found to be tendinopathic with some subluxation and had a concomitant LHB tenotomy. The subscapularis was found to be intact, and the supraspinatus and infraspinatus were both irreparably torn despite attempted soft tissue releases. After decompression, the planned arthroscopic superior capsular reconstruction was performed with three glenoid anchors, a lateral knotless double row, and a posterior margin convergence. Post-operative rehabilitation followed a standardized protocol, with 8 weeks in a sling before an active range of motion and 16 weeks before any light resisted exercises. There were no peri-operative complications after either procedure. 

The latest follow-up was four years and seven months after the right SCR and three years and nine months after the left SCR. He noted almost complete pain relief in both shoulders and restored active forward elevation and external rotation near full. He had returned to unrestricted activities of daily living as well as his normal occupation as a driver. 

Surgical technique

 The surgical technique for arthroscopic superior capsular reconstruction followed a defined set of steps. Procedures were done with the patient positioned on a beach chair. At the same time, the arm is held securely and safely using a supporting arm device that allows the arm to be moved in any direction during the procedure, under general anesthetic, and after an interscalene block. We started the diagnostic arthroscopy while keeping the pump pressure low (30-35 mmHg initially) and maintaining meticulous hemostasis with bipolar electrocautery throughout. The initial anterior working portal was kept high to aid in graft management later in the subacromial space. Once intra-articular pathology was assessed and treated, we moved to the subacromial space and localized our lateral portal more posteriorly than the usual “50-yard line” to keep more working room for the large anterolateral portal later. After an acromioplasty as necessary, while preserving the coracoacromial ligament, if possible, we moved to this posterolateral portal as the main viewing portal. 

 Releases around the rotator cuff are then performed to confirm irreparability, including anterior and posterior slides. Once the need for an SCR has been confirmed, bone preparation on the superior glenoid and proximal humeral footprint is accomplished with a combination of ring curettes, rasps, and shavers. Small marrow venting holes on the proximal humerus are also now created. A spinal needle then localizes the glenoid anchors (Arthrex 3 mm BioComposite suture anchors). We often used three anchors (Anterior to clavicle/Neviaser (Supraspinatus Fossa)/Posterior to acromion). The medial row humeral anchors (Arthrex 4.75 mm SwiveLock loaded with Fiber and Tiger Tape sutures, respectively) are also localized by spinal needle just off the acromial border. 

Graft sizing is critical and is done by positioning the arm in about 40 degrees of abduction, neutral rotation, and with minimal traction applied. Measurements for the graft size are made between all the anchors and marked on the graft, leaving a 10 mm border medially, anteriorly, posteriorly, and a 15 mm border laterally. A bone block and metal ruler as a stiff edge aids in cutting the graft, and medially the corners are beveled slightly to aid in graft insertion. We then punch lateral holes in the graft with a previously used SwiveLock driver to aid in sliding the thicker tap sutures laterally. 

Graft insertion is then accomplished by turning the pump back on and establish a view again from the posterolateral portal. A sterile towel is placed over the lateral arm to allow the graft to rest without sliding or touching the skin flora. The sutures are then sequentially retrieved from the joint out the anterolateral portal and shuttled through the graft, starting with the lower Fiber Tape and Tiger Tape sutures from the humeral anchors through their punched holes. The individual Suture tape limbs from the glenoid anchors passed with a Scorpion needle. The graft is then folded medially and held with a grasper, and introduced into the joint through the anterolateral portal. The assistants maintain gentle tension and remove slack from the passed sutures. 

**Figure 2:**
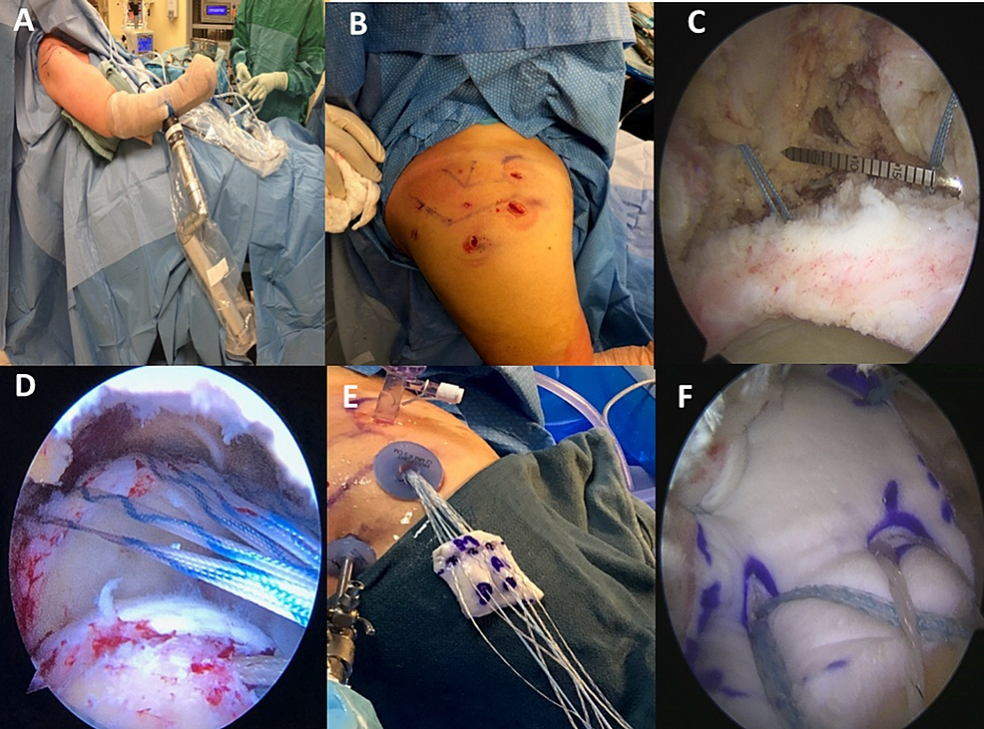
Selective clinical images showing some key points of our standard arthroscopic superior capsular reconstruction surgical technique. (A) The patient is positioned on a beach chair. At the same time, the arm is held securely and safely using a supporting arm device that allows the arm to be maintained in any position during the procedure. (B) The initial anterior working portal is kept high. In contrast, the lateral portal is positioned further posteriorly than the usual “50-yard line” to keep more working space for the large anterolateral portal. The small portals anterior to the clavicle, the supraspinatus fossa (Neviaser portal), and posterior to acromion are used to pass the glenoid suture anchors. (C) Graft sizing is critical and is done by positioning the arm in about 40 degrees of abduction, neutral rotation, and with minimal traction applied. Graft size measurements are made between all the anchors. (D) Sutures are retrieved sequentially from the joint through the anterolateral portal to be shuttled through the graft. (E) A sterile towel is placed on the lateral aspect of the arm to allow the graft to rest without sliding or touching the skin flora. (F) The graft is fixed laterally using a standard double row knotless suture bridge technique, and posterior margin convergence between native capsule/rotator cuff tissue and the graft is performed.

At this point, the glenoid sutures are parked sequentially in their respective portals of insertion, and the humeral sutures are parked in the anterior and posterior portals, respectively. The anterolateral portal is then free for knot tying on the glenoid side. If available, these tails are kept long for later incorporation of any residual stump of native rotator cuff tissue; otherwise, they are cut now. Lateral fixation proceeds with a standard double row knotless suture bridge technique, incorporating anterior and posterior dog-ear prophylaxis stitches. Finally, the posterior margin convergence between the native capsule and rotator cuff tissue and the graft is performed with a Scorpion and individually tied sutures. If any anterolateral rotator interval or transverse humeral ligament tissue is available, then anterior margin convergence to add to graft fixation is performed (Figure 2).

Postoperative rehabilitation is guided by a standardized protocol that includes four phases. The first phase (0-4 weeks) is the protection phase, during which patients are advised to keep the upper arm and elbow close to their side using a sling. Neck, elbow, wrist, and hand exercises are encouraged. The phase of controlled motion (second phase) is then initiated and lasts eight weeks. Patients are allowed to begin the closed chain pendulum exercise and shoulder passive range of motion either manually or with the help of a therapist. The third phase (8-16 weeks) is the progressive motion phase in which patients are allowed to start closed chain active-assisted range of motion and isometric strengthening exercises. Finally, the strengthening phase(> 16 weeks) is started, during which the patients are encouraged to build muscle strength and endurance through resistive strengthening and stretching exercises. 

## Discussion

Currently, the existing ideal indication for SCR is a patient with unbearable discomfort and/or intolerable dysfunction who has undergone unsuccessful non-surgical treatment and presents with massive and irreparable rotator cuff tears with minimal arthropathy, an intact or repairable tendon of the subscapularis, and an efficient deltoid muscle [4]. Additionally, those with pseudo-paralysis with combined superior glenohumeral instability might not be considered ideal for arthroplasty [1]. In contrast, patients with moderate to severe rotator cuff arthropathy (caution with Hamada 3, contraindicated with 4 and 5) of the shoulder joint are not considered potential candidates for this surgical intervention. These patients are generally better suited for shoulder arthroplasty. Likewise, patients with subscapularis tears unless the subscapularis can be restored at the time of the surgery [5]. Finally, patients with significant medical comorbidities or inadequate bone quality, as well as those reluctant to adhere to post-surgical treatment conditions, are not ideal candidates for SCR [6].

Theoretically, this surgical technique works by giving the shoulder joint an inactive restraint to excessive translation of the humeral head, thus improving the force couples of the defective rotator cuff [7]. In addition, reconstruction of the superior capsule stabilizes the superior aspect of the shoulder and thus improves the movements and functionality of the joint [8].

Despite the popularity of SCR, the literature on the clinical outcomes of the procedure is limited [9]. Recently, some authors have presented a case series of 100 patients who underwent this surgery [10]. The authors found that 93% of patients had an integrated graft, 94% of postoperative patients quickly returned to their initial tasks, and 100% of them participated in recreational sports [10]. These findings are consistent and supported by other researchers [11]. However, there is also evidence of graft failure from some studies in different settings, which could be due to differences in patient selection, graft preparation, or graft material. Studies are needed to assess the etiology of such graft failure [4].

## Conclusions

In our case, the patient had fully recovered from the procedure and returned to work without any restriction or complaint of either shoulder. We believe that performing bilateral SCR is a safe and reliable treatment option for young patients with bilateral irreparable massive rotator cuff tears. It provides a promising and excellent joint preserving treatment alternative with lower postoperative restriction than reverse shoulder arthroplasty.
